# Treatment and outcome analysis of patients with ruptured distal anterior cerebral artery aneurysms: a multicenter real-world study

**DOI:** 10.3389/fneur.2024.1329142

**Published:** 2024-02-26

**Authors:** Xiaowei Zhu, Zhen He, Zhuolin Wu, Yang Li, Yan Zhao, Bangyue Wang, Nai Zhang, Qiang Huang, Tao Yang, Minghao Yang, Jia Li, Xinyu Yang, Yanzhou Wang, Zhongyuan Zhang

**Affiliations:** ^1^Department of Neurosurgery, Tianjin Medical University General Hospital, Tianjin, China; ^2^Department of Neurosurgery, Yangquan First People's Hospital, Yangquan, Shanxi, China; ^3^Department of Neurosurgery, People's Hospital of Zunhua, Zunhua, Hebei, China; ^4^Department of Neurosurgery, Cangzhou Central Hospital of Hebei, Cangzhou, Hebei, China

**Keywords:** DACAA, complications, clipping, coiling, follow-up, prognostic outcomes

## Abstract

**Objective:**

To reveal the safety and efficacy of clipping and coiling in patients with ruptured distal anterior cerebral artery aneurysms (DACAA) and to calculate the risk factors affecting the two-year survival rate in follow-up patients.

**Methods:**

A retrospective study was conducted on the data of 140 patients (21 were lost to follow-up) with DACAA rupture who were treated by neurosurgery at 12 medical centers over a 2-year period, from January 2017 to December 2020. Univariate analysis was used to examine factors contributing to poor patient prognosis and to compare the prognosis of coiling and clipping treatments. Survival analysis was employed to compare survival rates between coiling and clipping, and risk factors affecting patient survival were analyzed using multivariate Cox regression analysis.

**Results:**

Out of 140 patients with ruptured DACAA, 80 (57.1%) were male, and 60 (42.9%) were female. A total of 111 (79.3%) patients were classified under Hunt-Hess scale grades I-III, while 95 (67.9%) were graded I-III according to the WFNs classification. Among them, 63 (45%) were treated with clipping, and 77 (55%) underwent coiling. Within 2 years of discharge from the hospital, 31 (59.6%) patients who underwent clipping and 54 (80.6%) who underwent coiling had a good prognosis. Multivariate Cox regression analysis revealed that only WFNs classification (I-III) was a protective factor influencing the 2-year survival of patients with ruptured DACAA.

**Conclusion:**

In the reality of medical practice, neurosurgeons are more likely to choose clipping as the treatment for cases with WFNs classification than or equal to III. There was no difference between clipping and coiling in the two-year prognosis at discharge. High priority should be given to DACAA cases with WFNs grading (I-III), as better outcomes can be achieved. The sample size will continue to be enlarged in the future to obtain more accurate findings. Abstracts for reviews, technical notes, and historical vignettes do not need to be separated into sections. They should begin with a clear statement of the paper’s purpose followed by appropriate details that support the authors’ conclusion(s).

## Introduction

DACAA is an aneurysm located on the anterior cerebral artery and its branches distal to the anterior communicating artery. DACAA accounts for a relatively small proportion of intracranial aneurysms, ranging from 1.5 to 9.0% as reported in the literature (1, 2). It is relatively rare in clinical practice. Lehecka et al. (3) classified them into A2 segmental aneurysms (below the knee of the corpus callosum and its frontal base branches), A3 segmental aneurysms (knee segment), and A4-5 segmental aneurysms (superior segment of the corpus callosum or distal branches such as the marginal callosal artery). In addition to causing subarachnoid hemorrhage, DACAA rupture is also highly associated with the formation of intracranial hematomas, with an incidence reported in the literature as high as 53%. The presence of intracranial hematoma in cases of DACAA rupture usually results in a more severe clinical grading compared to other types of aneurysms (4).

The treatment of DACAA is divided into two types: microsurgical and endovascular. Compared to intracranial aneurysms in other locations, DACAA is more challenging and carries a higher surgical risk, resulting in poorer treatment outcomes. As a result, it is often referred to as a malignant aneurysm. This difficulty primarily arises from the limited surgical field of view, challenges in controlling the parent arteries, and the tight adhesions between the aneurysm sac and the cingulate gyrus (5). Additionally, endovascular treatment can be challenging, with some authors reporting a higher incidence of intraoperative rupture and perforation for DACAA compared to other aneurysms, as well as a higher rate of unsuccessful interventional embolization due to its smaller size and more distant location (6, 7). The optimal treatment strategy for DACAA remains controversial, primarily due to the inherent challenges of both craniotomy clipping and coiling treatment.

The largest sample size in current studies on DACAA comes from a study by Martin Lehecka et al. (4), which included 501 cases of DACAA in two Finnish neurosurgical centers. However, no large sample data have been reported for DACAA in developing countries. Based on this background, our study includes data from patients with ruptured DACAA who were surgically treated in several Level IIIA hospitals in North China and followed up over the long term. The objective is to analyze the epidemiological characteristics and prognosis of such patients and to provide a basis for the diagnosis, treatment, and prognosis prediction of DACAA in developing countries.

## Methods

### Inclusion criteria and exclusion criteria

A retrospective study was conducted on the data of 140 patients (21 lost to follow-up) with DACAA rupture who were treated by neurosurgery at 12 Level IIIA medical centers in North China, including Tianjin Medical University General Hospital, over a 2-year period, from January 2017 to December 2020.

Inclusion criteria: (1) DACA ruptured aneurysm clearly diagnosed by cranial computed tomography angiogram (CTA), Magnetic resonance angiography (MRA), digital-subtraction angiography (DSA); (2) No contraindications to craniotomy and endovascular treatment; (3) Patients and their families voluntarily accepted craniotomy or endovascular treatment and signed the consent form to have treatment in the partner hospital of medical centers. Exclusion criteria: (1) patients chose conservative treatment, gave up treatment, or died before treatment; (2) no aneurysm treatment was performed in the partner hospital of medical centers.

### Characteristics of DACAA

DACAA was classified into four categories based on the longest diameter d of the aneurysm body (8): small aneurysms (d < 5 mm), medium-sized aneurysms (5 mm ≤ d < 15 mm), large aneurysms (15 mm ≤ d < 25 mm), and giant aneurysms (d ≥ 25 mm).

### Treatment modality

The treatment modalities are divided into microsurgical and endovascular treatments. The timing of surgery defines early aneurysm surgery as surgery performed within 3 days after the onset of subarachnoid hemorrhage, intermediate surgery as surgery performed from day 4 to day 7, and late surgery as surgery performed after 7 days (9).

### Clinical prognosis assessment

Clinical outcomes were assessed based on the survival rate, mortality rate, living status of follow-up patients at discharge, 1 month, 3 months, 6 months, 1 year, and 2 years after hospital discharge. We also used the Modified Ranking Scale (MRS) to evaluate prognosis, with a good prognosis graded as MRS scores 0–2 and a poor prognosis graded as MRS scores 3–6. Additionally, we employed multiple regression analysis to investigate the risk factors associated with poor outcomes.

### Statistical treatment

The statistical data were summarized, organized, and transformed into a data scale using Excel software. For the analysis of measurement data and ordered categorical data, we selected the rank sum test or T-test. For the analysis of count data, we chose Fisher’s exact test or the chi-square test. The chi-square test was used for data with categorical variables, while the rank sum test was used for continuous variables. Statistical analysis was performed using SPSS software, and statistical significance was defined as *p* < 0.05 for group data. Survival analysis of the DACAA patient population was conducted using Cox regression. Data were analyzed by using the statistical software of R studio (2022-05-20, vision) and SPSS (26.0.0.0, x64 vision). The R package was used for survival analysis and correlation analysis.

## Results

### Patient characteristics

Patient characteristics are presented in Table 1. Out of the 140 patients with ruptured DACAA, 80 (57.1%) were male, and 60 (42.9%) were female, with a mean age of 55.21 ± 9.36 years. The top three vascular risk factors for ruptured DACAA aneurysms were hypertension, a history of drinking, and a history of smoking. Among the patients, 111 (79.3%) were classified under Hunt-Hess grades I-III. Regarding treatment, 63 patients (45%) underwent clipping, while 77 patients (55%) received endovascular treatment. The mean follow-up time was 37.75 ± 19.88 months. On admission, 131 patients (93.6%) presented with symptoms of headache and vomiting, and 88 discharged patients (62.9%) had a Glasgow Outcome Scale (GOS) score of 5. In terms of imaging features, simple subarachnoid hemorrhage was the most common, occurring in 103 cases (73.6%), followed by combined intracranial hematoma in 28 cases (20%), and combined ventricular hemorrhage and hydrocephalus in 15 cases (10.7%), with 6 cases having both combined intracranial hematoma and ventricular hemorrhage hydrocephalus. Ninety-six patients (68.6%) were treated within 3 days of admission, 40 patients (28.6%) were treated within 4–7 days of admission, and 4 (2.8%) were treated after 7 days.

**Table 1:** Baseline characteristics.

Age, mean ± SD	55.21 ± 9.36	Preoperative symptoms	
Sex		Headache and vomiting	131 (93.6%)
Male, n (%)	80 (57.1%)	Movement disorders	37 (26.4%)
Female, n (%)	60 (42.9%)	Epilepsy	1 (0.7%)
Vascular risk factors		GOS score	
Hypertension	74 (52.9%)	1	5 (3.6%)
Diabetes	3 (2.1%)	2	15 (10.7%)
IS history	8 (5.7%)	3	15 (10.7%)
HS history	6 (4.3%)	4	17 (12.1%)
Alcohol history	20 (14.3%)	5	88 (62.9%)
Tobacco history	27 (19.3%)	Medical image features	
WFNs grade		SAH only	103 (73.6%)
I-III	95 (67.9%)	SAH with ICH	28 (20.0%)
IV–V	45 (32.1%)	SAH with IVH, hydrocephalus	15 (10.7%)
Hunt-Hess grade		Timing of admission surgery	
I-III	111 (79.3%)	Early surgery group	96 (68.6%)
IV–V	29 (20.7%)	Intermediate surgery group	40 (28.6%)
Fisher grade		Late surgery group	4 (2.8%)
I-II	90 (64.3%)		
III–IV	50 (35.7%)		
Treatment			
Clipping	63 (45%)		
Coiling	77 (55%)		
Time to procedure (months), mean ± SD	37.75 ± 19.88		

### Aneurysm characteristics

Aneurysm characteristics are presented in Table 2. Out of the 140 patients with ruptured DACAA, 63 (45%) had aneurysms located in segment A2, 76 (54.3%) in segment A3, and 1 (0.7%) in segment A4. Among the 140 aneurysms, 60 had complete descriptions of their diameters. The diameter of the aneurysmal neck averaged 2.79 mm, with 55 (91.7%) patients having a neck diameter of less than 5 mm. The mean diameter of the aneurysm dome was 4.53 mm, and in 45 cases (75%), the dome diameter was less than 5 mm. The mean vertical diameter of the aneurysm body was 3.34 mm, with 52 cases (86.7%) having a vertical diameter of less than 5 mm. The shape of the aneurysm was described in 115 cases, with 108 cases (93.9%) being saccular, 1 (0.9%) being fusiform, 3 (2.6%) being dissecting, and 3 (2.6%) being irregular. Among the 140 cases with aneurysms, 2 had multiple aneurysms, including one case with an aneurysm at segment A2 on the right side combined with microscopic aneurysms of the branch at A3 on the left side, and another case combined with a contralateral posterior communicating artery aneurysm. Additionally, 2 cases were associated with moyamoya disease, 1 case with anterior cranial fossa arteriovenous fistula, and 1 case with ipsilateral middle cerebral artery occlusion with localized moyamoya angiogenesis.

**Table 2:** Aneurysm characteristics of DACA.

Aneurysm position of DACA (*n* = 140)	
A2	63 (45%)
A3	76 (54.3%)
A4	1 (0.7%)
A5	0 (0%)
Aneurysm size of DACA (*n* = 60)	
Neck diameter, median (range)	2.79 (1–12)
Small (<5 mm)	55 (91.7%)
Medium (5–15 mm)	5 (8.3%)
Large (15–25 mm)	0
Giant (≥25 mm)	0
Dome diameter, median (range)	4.53 (1.4–15.31)
Small (<5 mm)	45 (75%)
Medium (5–15 mm)	13 (21.7%)
Large (15–25 mm)	2 (3.3%)
Giant (≥25 mm)	0
Vertical diameter, median (range)	3.34 (1.2–10)
Small (<5 mm)	52 (86.7%)
Medium (5–15 mm)	8 (13.3%)
Large (15–25 mm)	0
Giant (≥25 mm)	0
Aneurysm form of DACA (*n* = 115)	
Saccular	108 (93.9%)
Fusiform	1 (0.9%)
Dissecting	3 (2.6%)
Irregular	3 (2.6%)
Associated cerebrovascular findings (*n* = 140)	
Multiple aneurysm	2 (1.4%)
Moyamoya disease	2 (1.4%)
Arteriovenous fistula	1 (0.7%)
Vascular occlusion	1 (0.7%)

### Baseline between clipping and coiling groups

Out of the 140 patients with ruptured DACAA, 21 cases were lost to follow-up due to telephone errors. The remaining 119 cases were divided into the clipping and coiling groups based on the treatment modality, and the baseline characteristics and comparisons between the two groups are presented in Table 3.

**Table 3:** Baseline characteristics and comparison between the clipping and coiling groups.

Variable	Clipping group (*n* = 52)	Coiling group (*n* = 67)	*p* value
Loss of follow-up rate	11 (17.5%)	10 (13.0%)	0.461
Age, mean ± SD	55.98 ± 1.68	54.58 ± 0.90	0.115
Sex			
Male	30 (57.7%)	40 (59.7%)	0.825
Female	22 (42.3%)	27 (40.3%)	
Vascular risk factors			
Hypertension	27 (51.9%)	34 (50.7%)	0.899
Diabetes	2 (3.8%)	1 (1.5%)	0.417
IS history	4 (7.7%)	2 (3.0%)	0.244
HS history	3 (5.8%)	3 (4.5%)	0.749
Alcohol history	7 (13.5%)	11 (16.4%)	0.655
Tobacco history	8 (15.4%)	16 (23.9%)	0.252
WFNs grade			
I-III	31 (59.6%)	47 (70.1%)	0.230
IV–V	21 (40.4%)	20 (29.9%)	
Hunt-Hess grade			
I-III	37 (71.2%)	55 (82.1%)	0.158
IV–V	15 (28.8%)	12 (17.9%)	
Fisher grade			
I-II	26 (50.0%)	48 (71.6%)	0.016
III–IV	26 (50.0%)	19 (28.4%)	
Preoperative re-rupture	0 (0.0%)	3 (4.5%)	0.122
SAH with ICH	12 (23.1%)	11 (16.4%)	0.362
Hydrocephalus	5 (9.6%)	9 (13.4%)	0.521
Location of aneurysm			
A2	25 (48.1%)	28 (41.8%)	0.560
A3	27 (51.9%)	38 (56.7%)	
A4	0 (0.0%)	1 (1.5%)	
A5	0 (0.0%)	0 (0.0%)	
Timing of admission surgery			
Early surgery group	31 (59.6%)	52 (77.6%)	0.082
Intermediate surgery group	18 (34.6%)	14 (20.9%)	
Late surgery group	3 (5.8%)	1 (1.5%)	
Time to procedure (months), mean ± SD	37.06 ± 3.04	38.28 ± 2.24	0.100

In the clipping group, which included 52 cases (17.5% lost to follow-up), the mean age was 55.98 ± 1.68 years, with 30 males (57.7%). The coiling group, comprising 67 cases (13% lost to follow-up), had a mean age of 54.58 ± 0.90 years, with 40 males (59.7%). In the clipping group, no cases experienced preoperative re-rupture of the aneurysm. There were 12 cases (23.1%) with preoperative combined intracranial hematoma and 5 cases (9.6%) with preoperative combined hydrocephalus.

In the coiling group, 3 cases (4.5%) experienced preoperative re-rupture of the aneurysm, 11 cases (16.4%) had preoperative intracranial hematoma, and 9 cases (13.4%) had preoperative hydrocephalus. In the clipping group, 27 cases (51.9%) were located in segment A3, while in the coiling group, 38 cases (56.7%) were located in A3. In terms of timing, 31 cases (59.6%) in the clipping group underwent early surgery, while in the coiling group, 52 cases (77.6%) received early surgery. The mean follow-up time was 37.06 ± 3.04 months in the clipping group and 38.28 ± 2.24 months in the coiling group.

### Prognostic analysis of the follow-up between the clipping and coiling groups

The prognostic analysis of outcomes in the clipping and coiling groups is presented in Table 4. Multiple postoperative complications could occur in the same patients. Out of the 119 patients with ruptured DACAA, 87 (73.1%) had no complications, while 32 cases (26.9%) experienced periprocedural complications. In the clipping group, which consisted of 52 cases, 35 (67.3%) had no postoperative complications, while 17 cases (32.7%) experienced periprocedural complications. Among these cases, 3 had intraoperative re-rupture of the aneurysm (only one led to postoperative rebleeding), 4 had postoperative rebleeding, 8 had intracranial infections, 5 had postoperative cerebral infarctions, and 3 experienced paralysis. In the coiling group, comprising 67 cases, 52 (77.6%) had no postoperative complications, while 15 cases (22.4%) had periprocedural complications. There were no intraoperative aneurysm ruptures in this group. Among these cases, 2 had postoperative rebleeding, 4 had intracranial infections, 2 had urinary infections, 5 had postoperative cerebral infarctions, 1 had postoperative hydrocephalus treated with ventricular puncture and drainage, 3 had postoperative paralysis, and 1 experienced postoperative Guillain-Barré syndrome. It’s worth noting that postoperative cerebral infarction did not necessarily cause postoperative paralysis, and postoperative paralysis was not exclusively caused by postoperative cerebral infarction. Additionally, one case that initially underwent coiling treatment was converted to clipping treatment due to treatment failure.

**Table 4:** Outcomes compared between the clipping and coiling groups.

Outcome	Clipping group (*n* = 52)	Coiling group (*n* = 67)	*p* value
Procedure-related complication			
No	35 (67.3%)	52 (77.6%)	0.209
Intraoperative rupture	3 (5.8%)	0 (0%)	0.046
Postoperative hemorrhage	4 (7.7%)	2 (3.0%)	0.244
Intracranial infection	8 (15.4%)	4 (6.0%)	0.091
Urinary infection	0 (0.0%)	2 (3.0%)	0.209
Infarction	5 (9.6%)	5 (7.5%)	0.675
Hydrocephalus	0 (0.0%)	1 (1.5%)	0.376
Paralysis	3 (5.8%)	3 (4.5%)	0.749
Occlusion grade after treatment			
Total occlusion	50 (96.2%)	62 (92.5%)	0.355
Neck remnant	1 (1.9%)	4 (6.0%)	
Tumor remnant	0 (0.0%)	1 (1.5%)	
Wrapping	1 (1.9%)	0 (0.0%)	
Prognosis (GOS score)			
Favorable (4–5)	31 (59.6%)	55 (82.1%)	0.007
Unfavorable (1–3)	21 (40.4%)	12 (17.9%)	
Favorable outcome (MRS ≤ 2)			
At discharge	30 (57.7%)	45 (67.2%)	0.228
6 months	32 (61.5%)	54 (80.6%)	0.021
2 years	31 (59.6%)	54 (80.6%)	0.012
Death			
30 days	9 (17.3%)	6 (9.0%)	0.173
3 months	9 (17.3%)	6 (9.0%)	0.173
6 months	10 (19.2%)	6 (9.0%)	0.103
1 years	10 (19.2%)	7 (10.4%)	0.174
2 years	12 (23.1%)	9 (13.4%)	0.171
Follow-up period			
Hydrocephalus	3 (5.8%)	0 (0%)	0.046
Epilepsy	3 (5.8%)	1 (1.5%)	0.199
Recurrence	2 (3.8%)	0 (0%)	0.105

In the clipping group, regarding the rate of aneurysm occlusion, 50 cases (96.2%) achieved total occlusion, 1 case had remnants of the aneurysm neck, and 1 case was unable to clip the aneurysm and underwent wrapping. In the coiling group, 62 cases (92.5%) achieved total occlusion, 4 cases had remnants of the aneurysm neck, and 1 case had remnants of the aneurysm body. At discharge, the prognosis was good in 30 cases (57.7%) in the clipping group and in 45 cases (67.2%) in the coiling group. Within 2 years of discharge, 12 cases (23.1%) in the clipping group and 9 cases (13.4%) in the coiling group passed away. During the 2-year follow-up period, the clipping group experienced 3 cases of hydrocephalus, 3 cases of epilepsy, and 2 cases of aneurysm recurrence, while the coiling group had 1 case of epilepsy, with no occurrences of hydrocephalus or aneurysm recurrence.

### Prognosis analysis of 119 follow-up patients in 2 years after leaving the hospital

The prognostic outcomes of the 119 follow-up patients at 2 years after leaving the hospital were analyzed based on MRS scores for the good and poor prognosis groups (refer to Table 5). In the good prognosis group, there were 85 cases (71.4%) with a mean age of 55.22 ± 0.84 years, including 44 cases (51.8%) of males. The poor prognosis group consisted of 34 cases (28.6%) with a mean age of 55.12 ± 2.32 years, with 26 cases (76.5%) being males. Among the good prognosis group, 39 cases (45.9%) had combined hypertension, while in the poor prognosis group, 22 cases (64.7%) had combined hypertension.

**Table 5:** The comparison of good (mRS ≤ 2) and poor (mRS 3–6) prognosis group at 2 years.

Variable	Good (*n* = 85)	Poor (*n* = 34)	*p* value
Age, mean ± SD	55.22 ± 0.84	55.12 ± 2.32	0.133
Sex			
Male	44 (51.8%)	26 (76.5%)	0.013
Female	41 (48.2%)	8 (23.5%)	
Vascular risk factors			
Hypertension	39 (45.9%)	22 (64.7%)	0.063
Diabetes	2 (2.4%)	1 (2.9%)	0.853
IS history	3 (3.5%)	3 (8.8%)	0.233
HS history	3 (3.5%)	3 (8.8%)	0.233
Alcohol history	14 (16.5%)	4 (11.8%)	0.517
Tobacco history	17 (20.0%)	7 (20.6%)	0.942
WFNs grade			
I-III	72 (84.7%)	6 (17.6%)	<0.001
IV–V	13 (15.3%)	28 (82.4%)	
Hunt-Hess grade			
I-III	79 (92.9%)	13 (38.2%)	<0.001
IV–V	6 (7.1%)	21 (61.8%)	
Fisher grade			
I-II	61 (71.8%)	13 (38.2%)	0.001
III–IV	24 (28.2%)	21 (61.8%)	
Location of aneurysm			
A2	37 (43.5%)	16 (47.1%)	0.782
A3	47 (55.3%)	18 (52.9%)	
A4	1 (1.2%)	0 (0.0%)	
A5	0 (0.0%)	0 (0.0%)	
Timing of admission surgery			
Early surgery group	53 (62.4%)	30 (88.2%)	0.018
Intermediate surgery group	28 (32.9%)	4 (11.8%)	
Late surgery group	4 (4.7%)	0 (0.0%)	
Time to procedure (months), mean ± SD	44.86 ± 1.48	19.97 ± 3.77	<0.001
Preoperative re-rupture			
Yes	0 (0.0%)	3 (8.8%)	0.006
No	85 (100.0%)	31 (91.2%)	
SAH with ICH			
Yes	9 (10.6%)	14 (41.2%)	<0.001
No	76 (89.4%)	20 (58.8%)	
Hydrocephalus			
Yes	5 (5.9%)	9 (26.5%)	0.002
No	80 (94.1%)	25 (73.5%)	
Treatment			
Clipping	31 (36.5%)	21 (61.8%)	0.012
Coiling	54 (63.5%)	13 (38.2%)	
Procedure-related complication			
Yes	16 (18.8%)	16 (47.1%)	0.002
No	69 (81.2%)	18 (52.9%)	

In the good prognosis group, there were 53 cases (62.4%) that underwent early surgery, with no cases of preoperative re-rupture of the aneurysm, 9 cases (10.6%) of preoperative combined intracranial hematoma, 5 cases (5.9%) of preoperative combined hydrocephalus, 31 cases (36.5%) that received clipping, and 16 cases (18.8%) that experienced postoperative complications. In the poor prognosis group, 30 cases (88.2%) underwent early surgery, 3 cases (8.8%) had preoperative re-rupture of the aneurysm, 14 cases (41.2%) had preoperative combined intracranial hematoma, 9 cases (26.5%) had preoperative combined hydrocephalus, 21 cases (61.8%) received clipping, and 16 cases (47.1%) had postoperative complications.

### Univariate and multivariate analysis of risk factors affecting patient survival

The univariate and multivariate analyses affecting patient survival are presented in Tables 6, 7. A total of 17 factors were included as independent variables to analyze the risk factors affecting the survival of the patients: age, gender (female), hypertension, diabetes mellitus, history of IS (history of ischemic stroke), history of HS (history of hemorrhagic stroke), drinking history, smoking history, WFNs classification (I-III), Hunt-Hess classification (I-III), aneurysm location, timing of admission surgery, preoperative re-rupture of aneurysm, preoperative combined intracranial hematoma, preoperative combined hydrocephalus, and treatment modality.

**Table 6:** Univariate Cox regression.

Characteristics	Hazard ratio (95%CI)	*p* value
Age	1.248 (0.809–1.924)	0.317
Sex (Female)	0.395 (0.178–0.877)	0.023
Hypertension	1.662 (0.816–3.385)	0.162
Diabetes	0.846 (0.114–6.297)	0.871
IS history	2.355 (0.713–7.787)	0.160
HS history	0.495 (0.067–3.630)	0.489
Alcohol history	0.525 (0.160–1.724)	0.288
Tobacco history	0.908 (0.375–2.201)	0.832
WFNs grade (I-III)	0.117 (0.050–0.270)	<0.001
Hunt-Hess grade (I-III)	0.151 (0.075–0.305)	<0.001
Fisher grade (I-II)	0.246 (0.119–0.509)	<0.001
Location of aneurysm	0.862 (0.441–1.687)	0.665
Timing of admission surgery	0.294 (0.107–0.808)	0.018
Preoperative re-rupture	4.045 (0.952–17.183)	0.058
SAH with ICH	2.617 (1.317–5.201)	0.006
Hydrocephalus	3.689 (0.844–16.12)	0.083
Treatment	0.457 (0.224–0.93)	0.031

**Table 7:** Multivariate Cox regression.

Characteristics	Hazard ratio (95%CI)	*p* value
Sex (Female)	0.603 (0.258–1.41)	0.243
WFNs grade (I-III)	0.23 (0.072–0.735)	0.013
Hunt-Hess grade (I-III)	0.66 (0.248–1.758)	0.406
Fisher grade (I-II)	0.603 (0.205–1.772)	0.358
Timing of admission surgery	0.585 (0.192–1.784)	0.346
SAH with ICH	0.734 (0.286–1.887)	0.521
Treatment	0.608 (0.29–1.272)	0.186

Univariate Cox regression analysis revealed that being female, having WFNs grading (I-III), Hunt-Hess grading (I-III), undergoing surgery at a specific timing of admission, experiencing preoperative combined intracranial hematoma, and receiving a particular treatment modality were protective factors affecting the 2-year survival of patients with ruptured DACAA.

Multivariate Cox regression analysis, however, demonstrated that only WFNs classification (I-III) (HR (95%CI): 0.209 (0.070–0.631), *p* = 0.005) was a significant protective factor affecting the 2-year survival of patients with ruptured DACAA, while the remaining factors did not reach statistical significance.

### Correlation analysis

**Figure 1:**
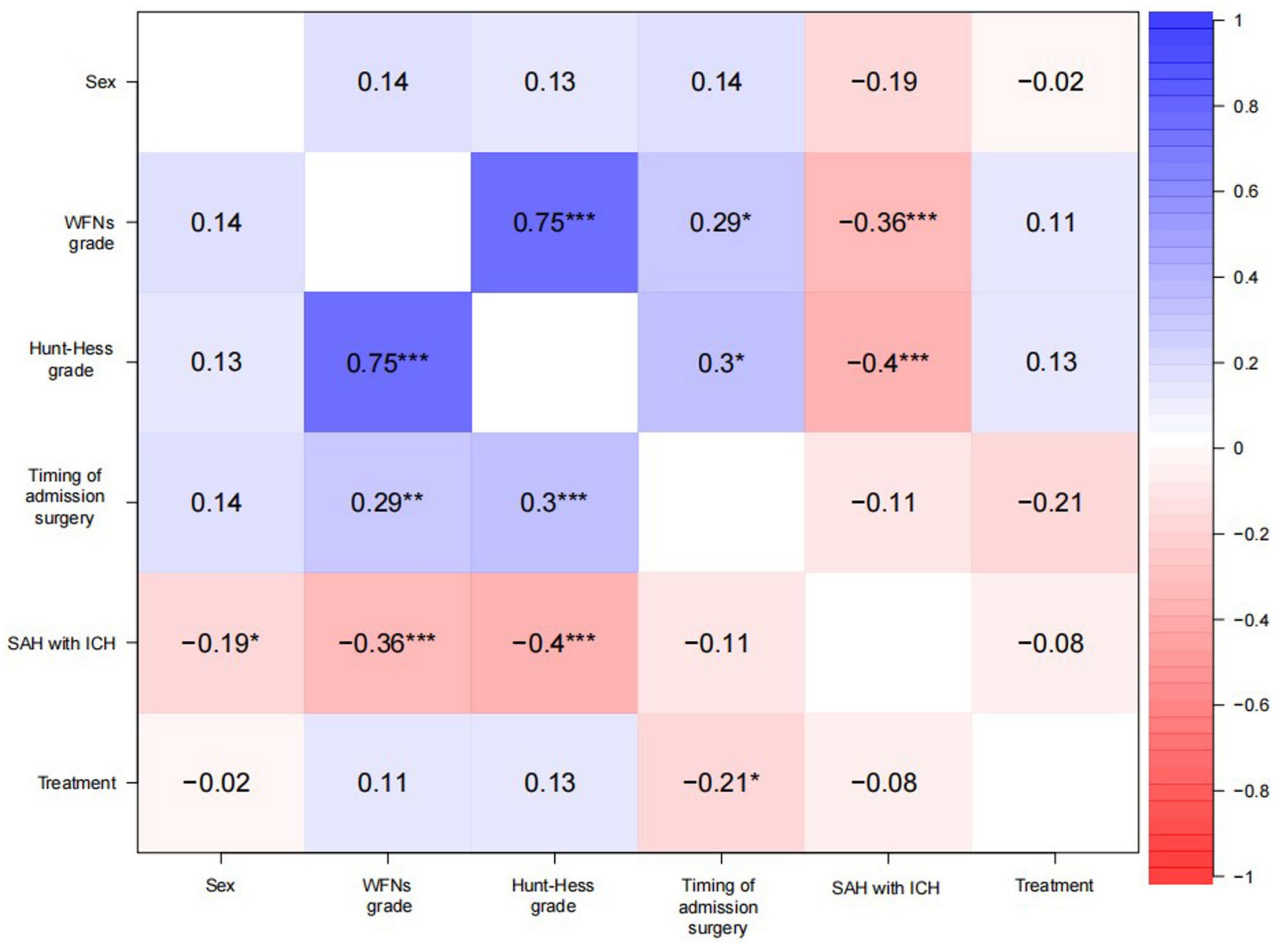
Heat map of correlation.

The correlation analysis explored the effects of the variables’ prior existence, where positive correlation values were greater than 0; negative correlation values were greater than 0 (Figure 1). There was no significance for the treatment, probably due to a negative correlation with the timing of surgery (−0.21, *p* < 0.05).

### Survival analysis curves

**Figure 2:**
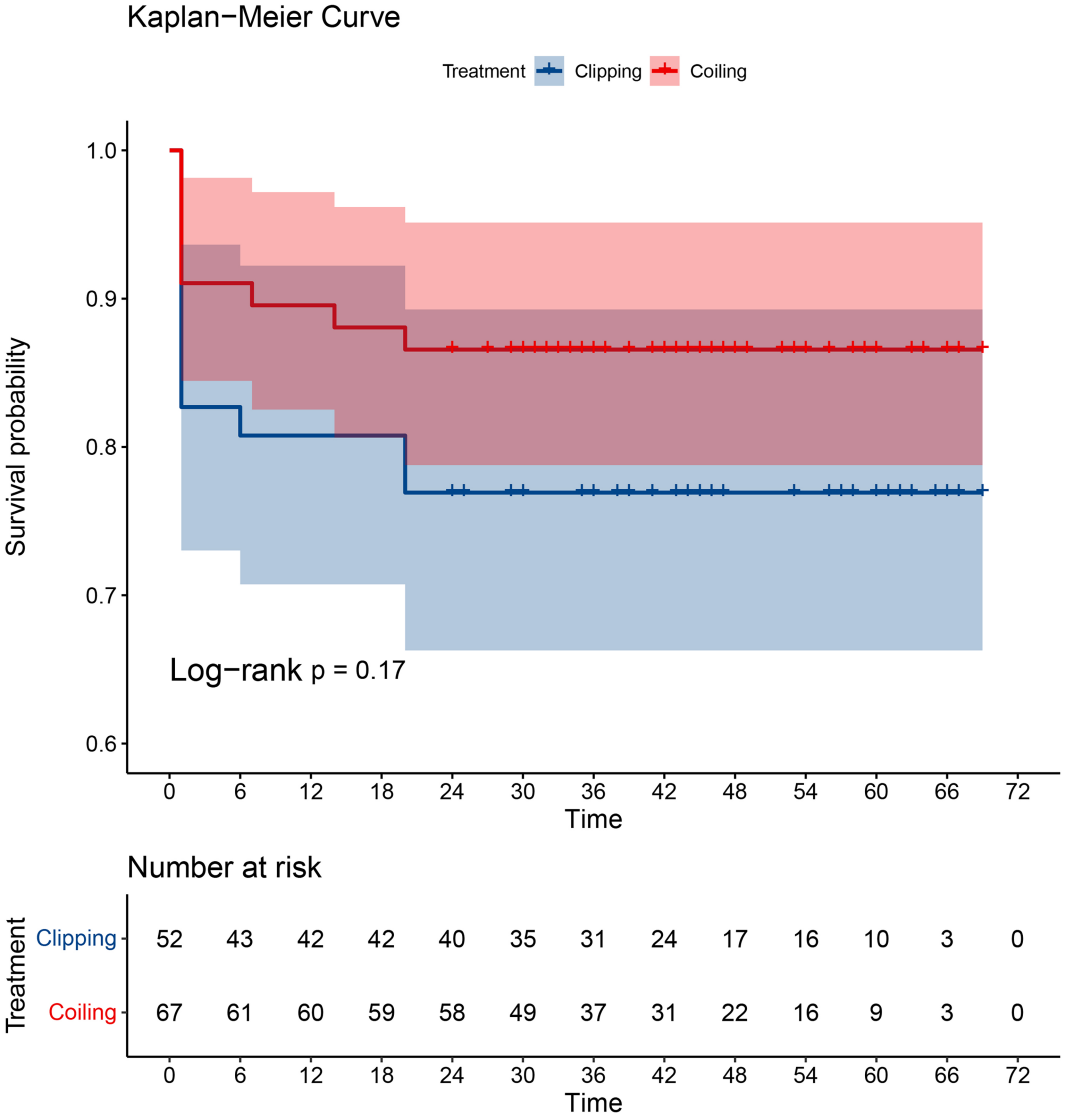
The K-M survival curves for the clipping group and the coiling group.

The Kaplan–Meier survival curves for the clipping group and the coiling group are displayed in Figure 2. It can be observed that there is no overlap between the survival curves of the two groups, and the survival rate of the coiling group appears to be better than that of the clipping group. However, the statistical test (log-rank) of the survival rates of the two groups yielded a value of *p* greater than 0.05, indicating that the difference in survival rates between the two groups was not statistically significant.

## Discussion

DACAA is exceptionally rare in clinical practice, with only a few case reports in the literature documenting more than 100 cases. For instance, Martin Lehecka et al. (4) assessed the outcomes of 277 patients with ruptured DACA aneurysms and 150 patients with unruptured DACA aneurysms over the past 25 years, averaging less than 20 cases per year. They reported treating only 12 cases of ruptured DACAA with coiling during that period. In contrast, our study evaluates the 2-year prognostic outcomes of ruptured DACAA over the past 4 years, including 63 patients treated with clipping and 77 patients treated with coiling, averaging 35 cases per year. As a result, our study currently boasts the largest sample size for endovascular treatment.

This is a retrospective, multicenter, real-world study conducted in North China to assess the safety and efficacy of clipping and coiling as treatment options for ruptured DACAA. The study also aims to examine the risk factors that influence the two-year survival of patients. By statistically analyzing data from 140 patients with surgically treated ruptured DACAA, we have observed that there is no significant difference in survival and aneurysm occlusion rates between the clipping and coiling groups. However, we have found that the prognosis of the coiling group is significantly better than that of the clipping group. Additionally, our analysis has identified WFNs classification (I-III) as a protective factor influencing the two-year prognostic outcomes of patients. Although DACAA is rare in clinical practice, its rupture often leads to more severe clinical grading, and the optimal treatment strategy remains a subject of controversy. This study offers valuable insights for making treatment choices for this type of aneurysm and highlights the relevant factors that impact patients’ prognosis and survival.

The literature reports that A3 segment aneurysms account for 69–72% of DACAA (3), whereas in our study, they accounted for 54.3%. Hemesniemi et al. (10) showed that the mean diameter of ruptured DACAA was 8.1 mm, while the mean diameter of 1,002 intracranial ruptured aneurysms at other sites was 11.3 mm. Ohno reported (11) 11 that the proportion of ruptured DACAA with a diameter of <5 mm was 67%. Typically, the size of ruptured DACAA is small, usually ranging from 5 to 8 mm (12), and more than 50% of ruptured aneurysms are <7 mm in diameter 4. In our study, the mean aneurysm neck diameter was 2.79 mm, with 91.7% of cases having a diameter < 5 mm. This indicates that DACAA tends to be smaller in size compared to other intracranial aneurysms.

There remains considerable controversy surrounding the treatment of DACAA today. The International Subarachnoid Aneurysm Trial (ISAT) has demonstrated that spring coil embolization leads to better outcomes for ruptured intracranial aneurysms compared to craniotomy clipping (13, 14). As a result of its simplicity and surgical outcomes that are no less favorable than clipping, coiling therapy has been widely adopted for aneurysm treatment in various centers. In our study, there was no difference in the choice of surgical treatment based on age, gender, vascular risk factors, WFNs grade, Hunt-Hess grade, preoperative re-rupture of the aneurysm, preoperative combined intracranial hematoma, preoperative combined hydrocephalus, location of the aneurysm, or timing of surgery. Interestingly, our study did not yield the results we originally anticipated, which would have suggested that patients with combined vascular risk factors were more likely to choose coiling treatment, and patients with preoperative combined intracranial hematoma were more likely to choose clipping treatment.

A study conducted by Thomas Metayer (15) involving 69 surgically treated DACAA cases showed no significant difference in postoperative complications between clipping and coiling therapies. Our analysis of the seven most common postoperative complications revealed that, for ruptured DACAA, although clipping was more likely to result in intraoperative rupture of the aneurysm compared to coiling, there was no difference between the two treatment modalities in terms of the overall rate of potential postoperative complications. Keston et al. (6) reported a postoperative rebleeding rate of about 7–16% with DACAA clipping therapy, which was consistent with our findings (7.7%). Furthermore, our study demonstrated that there was no significant difference between the two treatment modalities in terms of the postoperative bleeding rate. Postoperative intracranial infection was the most common complication in the clipping group, but there was no statistically significant difference between the two treatment modalities. The most common reason for the failure of endovascular treatment was the challenging placement of the distal artery or instability of the small aneurysm sac coil (16). In our study, only one case failed in the coiling treatment and subsequently underwent clipping treatment. Remarkably, 92.5% of the coiling patients exhibited complete aneurysm occlusion on immediate postoperative imaging results. There was no significant difference between the two treatment modalities in terms of aneurysm occlusion rate.

A meta-analysis conducted by Petr et al. (17) demonstrated that there was no significant difference in the impact of coiling and clipping on mortality in patients with DACAA. In our study, we found no significant difference in patient survival between the two treatment modalities at 30 days, 3 months, 6 months, 1 year, and 2 years after discharge from the hospital. We conducted survival analysis and compared survival rates between the two treatments based on patient follow-up time and final survival status. The results of our survival analysis indicate that there is no statistically significant difference in survival rates between the two treatment modalities (*p* > 0.05), despite the fact that the survival curve was higher in the coiling group compared to the clipping group. Therefore, we conclude that neither of the two treatment modalities significantly affects patient survival rates.

A study conducted by Hui et al. (18) concluded that there was no significant difference in the prognostic impact of clipping and coiling therapy on patients with DACAA. However, in patients with concomitant SAH, coiling therapy was associated with a better prognosis. Our results align with these findings, as we observed no difference in prognosis between coiling therapy and clipping at the time of discharge. However, we did find that the prognosis of coiling therapy was superior to that of clipping at 6 months and 2 years after discharge from the hospital. This is consistent with the results reported by Petr et al. (17). In comparison to the study by Hui et al., our research boasts a larger sample size and a longer follow-up period, which enhances the statistical significance of our findings. Therefore, we believe that for surviving patients, coiling therapy offers a more favorable prognosis compared to clipping therapy as they continue their recovery after leaving the hospital.

Previous studies have consistently shown that several factors, including age, Hunt-Hess grade at admission, intracranial hematoma, intraventricular hemorrhage, severe preoperative hydrocephalus, and pre-treatment rebleeding, are all significant predictors of poor prognosis in patients with DACAA, regardless of the treatment modality used, be it clipping or coiling therapy (16, 17, 19). In addition, Martin Lehecka et al. (4) reported that advanced age and higher Hunt-Hess classification (III-V) were risk factors for poor prognosis in ruptured DACAA. Yushiro Take’s study of 37 surgically treated DACAA cases also indicated that WFNs grade (IV-V) and intracranial hematoma were risk factors for poor prognosis (20). In light of these findings, we conducted univariate and multivariate Cox regression analyses on 17 factors that could potentially affect the survival status of patients with ruptured DACAA at the 2-year mark after hospital discharge. The results revealed that WFNs grade (I-III) was the only statistically significant protective factor affecting the 2-year survival of patients with ruptured DACAA. We hypothesize that a better WFNs grade reflects a better preoperative health status for the patient, increasing the likelihood of a favorable prognosis. The World Federation of Neurosurgical Societies grading scales (WFNs) are now considered the gold standard for the initial clinical assessment of patients with spontaneous subarachnoid hemorrhage upon hospital admission. Combining these results with our study findings, we conclude that the WFNs classification, which assesses aSAH based on the GCS score, serves as a more objective predictor of 2-year survival in patients with DACAA rupture compared to the Hunt-Hess classification.

### Limitations

This study is retrospective in nature, and it may not encompass all the relevant factors that could influence patient prognosis and survival. It’s important to note that 21 cases, accounting for 15% of the initial 140 patients, were lost to follow-up at the 2-year mark.

## Conclusion

Intraoperative rupture of aneurysms is relatively common in clipping therapy. There is no significant difference between clipping and coiling treatments in terms of survival and complete aneurysm occlusion rates in patients with DACAA WFNs classification (I-III) is identified as a protective factor affecting the 2-year survival of patients with ruptured DACAA. This study provides valuable insights for future treatment decisions and long-term prognosis prediction for patients with DACAA rupture in developing countries.

## Data availability statement

The raw data supporting the conclusions of this article will be made available by the authors, without undue reservation.

## Ethics statement

The studies involving humans were approved by the Ethics Committee of Tianjin Medical University General Hospital. The studies were conducted in accordance with the local legislation and institutional requirements. Written informed consent for participation was not required from the participants or the participants’ legal guardians/next of kin in accordance with the national legislation and institutional requirements.

## Author contributions

XZ: Data curation, Writing – original draft. ZH: Methodology, Writing – review & editing. ZW: Writing – review & editing. YL: Formal analysis, Writing – original draft. YZ: Methodology, Writing – review & editing. BW: Methodology, Writing – original draft. NZ: Data curation, Writing – review & editing. QH: Conceptualization, Writing – review & editing. TY: Formal analysis, Writing – original draft. MY: Writing – review & editing. JL: Writing – review & editing. XY: Visualization, Writing – review & editing. YW: Supervision, Writing – review & editing. ZZ: Validation, Writing – review & editing.
